# Pharmacotherapeutic management of insomnia and effects on sleep processes, neural plasticity, and brain systems modulating stress: A narrative review

**DOI:** 10.3389/fnins.2022.893015

**Published:** 2022-07-29

**Authors:** Laura Palagini, Carlotta Bianchini

**Affiliations:** ^1^Psychiatry Division, Department of Neuroscience and Rehabilitation, University of Ferrara, Ferrara, Italy; ^2^Department of Clinical and Experimental Medicine, University of Pisa, Pisa, Italy; ^3^Department of Medical, Pfizer Italia, Rome, Italy

**Keywords:** insomnia, GABAA receptors, hypnotic benzodiazepines, Z-drugs, sleep processes, brain plasticity, stress system

## Abstract

**Introduction:**

Insomnia is a stress-related sleep disorder, may favor a state of allostatic overload impairing brain neuroplasticity, stress immune and endocrine pathways, and may contribute to mental and physical disorders. In this framework, assessing and targeting insomnia is of importance.

**Aim:**

Since maladaptive neuroplasticity and allostatic overload are hypothesized to be related to GABAergic alterations, compounds targeting GABA may play a key role. Accordingly, the aim of this review was to discuss the effect of GABA_*A*_ receptor agonists, short-medium acting hypnotic benzodiazepines and the so called Z-drugs, at a molecular level.

**Method:**

Literature searches were done according to PRISMA guidelines. Several combinations of terms were used such as “hypnotic benzodiazepines” or “brotizolam,” or “lormetazepam” or “temazepam” or “triazolam” or “zolpidem” or “zopiclone” or “zaleplon” or “eszopiclone” and “insomnia” and “effects on sleep” and “effect on brain plasticity” and “effect on stress system”. Given the complexity and heterogeneity of existing literature, we ended up with a narrative review.

**Results:**

Among short-medium acting compounds, triazolam has been the most studied and may regulate the stress system at central and peripheral levels. Among Z-drugs eszopiclone may regulate the stress system. Some compounds may produce more “physiological” sleep such as brotizolam, triazolam, and eszopiclone and probably may not impair sleep processes and related neural plasticity. In particular, triazolam, eszopiclone, and zaleplon studied *in vivo* in animal models did not alter neuroplasticity.

**Conclusion:**

Current models of insomnia may lead us to revise the way in which we use hypnotic compounds in clinical practice. Specifically, compounds should target sleep processes, the stress system, and sustain neural plasticity. In this framework, among the short/medium acting hypnotic benzodiazepines, triazolam has been the most studied compound while among the Z-drugs eszopiclone has demonstrated interesting effects. Both offer potential new insight for treating insomnia.

## Introduction

Insomnia is the most frequent among sleep and psychiatric disorders and may affect more than 30% of the population in industrialized countries (American Psychiatric Association, 2013), with an overall increase during the COVID-19 pandemic in around 55% of the population (Morin and Carrier, 2021).

While sleep is essential for brain homeostasis, brain plasticity, and mental and physical health (Cirelli and Tononi, 2017; de Vivo et al., 2017; Ramar et al., 2021) chronic insomnia may favor a state of allostatic overload impairing neural plasticity, immune and endocrine pathways, and may contribute to mental and physical disorders (Fernandez-Mendoza and Vgontzas, 2013; Hertenstein et al., 2019; Lo Martire et al., 2020; Nobre et al., 2021; Van Someren, 2021).

Insomnia symptoms may hold detrimental daytime consequences and may be associated with more than twice the odds of having a comorbid condition including heart disease or hypertension, and has been strongly associated with neuropsychiatric and mental disorders including neurodegenerative diseases, depression, and anxiety, independently increasing suicidal risk (Pigeon et al., 2012; Fernandez-Mendoza and Vgontzas, 2013; Hertenstein et al., 2019; Nobre et al., 2021). Due to its chronicity, insomnia may substantially impair quality of life and global functioning.

Within this framework, assessing and targeting insomnia is of great importance. In particular recent studies demonstrated that pharmacological and psychological interventions for insomnia may impact favorably on the trajectory of psychiatric and medical disorders (Manber et al., 2011; Dallaspezia and Benedetti, 2015; Cosgrave et al., 2018; Geoffroy et al., 2018; Benard et al., 2019).

Recent guidelines for the diagnosis and management of insomnia disorder (Riemann et al., 2017; Sateia et al., 2017; Wilson et al., 2019; Palagini et al., 2020) recommend cognitive behavioral therapy for insomnia (CBT-I) as the first-line approach for chronic forms of insomnia. Among the available pharmacological options, gamma-aminobutyric acid (GABA)_*A*_ receptor agonists, such as benzodiazepines and *Z*-drugs including zolpidem, zaleplon zopiclone, and eszopiclone, have been used for decades in insomnia management (Riemann et al., 2017; Sateia et al., 2017; Wilson et al., 2019; Palagini et al., 2020), while the melatonin receptor agonists, ramelteon and prolonged-release melatonin 2 mg represent new important therapeutic options acting on circadian sleep regulation (Wilson et al., 2019; Palagini et al., 2020). Since the maladaptive neuroplasticity and allostatic overload hypothesized in chronic insomnia may be related to alterations in GABAergic neurotransmission, we can speculate that GABA_*A*_ receptor modulators, such as hypnotic benzodiazepines and *Z*-drugs, may be able to remodulate the changes in sleep architecture/neuronal structure and function induced by chronic insomnia.

Although meta-analyses have confirmed the clinical efficacy of short- and intermediate-acting benzodiazepines and *Z*-drugs for the short-term treatment (≤4 weeks) of insomnia (Pillai et al., 2017; Lynch et al., 2020; Chiu et al., 2021), many concerns have reported during the last few years about the use of *Z*-drugs, originally marketed as a safe alternative to benzodiazepines (Schifano et al., 2019). Accordingly, the aim of this review was to focus at a physiological level on the effect of benzodiazepines and *Z*-drugs on sleep regulation, brain plasticity, and the stress system, which are altered in chronic insomnia. To this end, we discussed the physiological aspects of GABA and GABA_*A*_ receptor activity on sleep, brain plasticity, and brain systems modulating stress, with potential implications for chronic insomnia pathophysiology. We then reviewed the available data on the effect of hypnotic benzodiazepines, on sleep, neuroplasticity and brain systems modulating stress with implications for insomnia treatment. We also discuss the available data on commonly prescribed benzodiazepines for chronic insomnia, which include the short- and intermediate-acting hypnotics (such as brotizolam, lormetazepam, temazepam, triazolam) and the *Z*-drugs (zolpidem, zopiclone, eszopiclone, zaleplon). Although flurazepam, oxazepam, lorazepam, and diazepam are widely used as hypnotics, they are also indicated for anxiety disorders and are not included in this review.

## Method

Literature searches were done according to PRISMA guidelines (Moher et al., 2009) using several combinations of search terms, including: “hypnotic benzodiazepines,” or “brotizolam,” or “lormetazepam,” or “temazepam,” or “triazolam,” or “zolpidem,” or “zopiclone,” or “zaleplon,” or “eszopiclone” and “insomnia” and “effects on sleep” and “effect on brain plasticity” and “effect on stress system.”

Inclusion criteria were experimental and clinical studies or review articles on animals and humans that assessed the effect of hypnotic benzodiazepines or *Z*-drugs on sleep pattern and regulation, sleep-related synaptic plasticity, and on brain circuits modulating stress. Review articles and studies that evaluated other sleep or other psychiatric disorders were excluded. However, given the complexity and heterogeneity of existing research, with most of the studies performed in the early 1980s showing varying methodological quality, we were unable to perform a systematic literature review and instead presented our findings as a narrative review.

As hypnotic benzodiazepines and *Z*-drugs act selectively on GABA_*A*_ receptors, we initially discussed the role of GABA and GABA_*A*_ receptors in sleep and brain systems modulating stress, with implications for chronic insomnia pathophysiology, while the second part of our review discussed the implications for insomnia treatment.

## Results

### GABAergic transmission, sleep, and brain systems modulating stress

GABA, a four-carbon non-proteinogenic amino acid, is the most important inhibitory neurotransmitter present in high concentrations in different areas of the mammalian brain. Thus, approximately 20–50% of all synapses are under GABAergic inhibitory control (Montaldo et al., 1984). GABA exerts its primary function at the synaptic level, where, by binding to pre/post-synaptic GABA_*A*_ receptors, it activates chloride ion channels and hyperpolarizes the cell membranes, thus modulating the threshold of neuronal excitability (Biggio et al., 1977; Study and Barker, 1981; Biggio, 1983; Zhu et al., 2018). GABA_*A*_ receptors are characterized by multiple allosteric binding sites, including those for benzodiazepines (Braestrup and Squires, 1977).

Ligands that interact with these binding sites induce a conformational change in GABA_*A*_ receptors, and thereby modulate GABA_*A*_-receptor functioning. Given the ubiquitous role of GABA as an inhibitory neurotransmitter and its widespread distribution in the brain, GABA_*A*_ receptors play a major role in regulating multiple neuronal systems, including those modulating stress response, sleep pattern, and circadian rhythms (Diana et al., 2014; Rashmi et al., 2018). Indeed, GABAergic neurons and other neurotransmitters play a major role in regulating the sleep-wake system at cortico-medullary pathways. GABAergic neurons may modulate both rapid eye movement (REM) and non-REM (NREM), particularly slow wave sleep (SWS) (Luppi et al., 2017), and, at the suprachiasmatic nucleus (SCN), modulate circadian rhythm (DeWoskin et al., 2015).

Moreover, activation of GABA_*A*_ receptors is implicated in the regulation of sleep, and GABAergic activity from the ventrolateral preoptic nucleus (VLPO) of the hypothalamus exerts an inhibitory control over the ascending arousal network. The latter is sustained by many wake promoting circuits including cholinergic basal forebrain, histaminergic tuberomammillary nucleus, serotonergic dorsal raphe, and noradrenaline producing locus coeruleus, with orexin neurons sending excitatory projection to the thalamus and neocortex (Mavanji et al., 2015). Moreover, GABAergic neurons make inhibitory connections with both orexin and ascending arousal systems (Ferrari et al., 2018).

Since GABA is involved in the synaptic plasticity changes of the retinohypothalamic tract-SCN synapses, it has been hypothesized that GABA may regulate the circadian rhythm by modulating both input and output of circadian oscillation in the SCN. This is important since sleep is essential for brain homeostasis and for synaptic renormalization according to the synaptic homeostasis hypothesis, which proposes that sleep has a functional role in synaptic plasticity through the sleep/wake cycle (Tononi and Cirelli, 2003, 2020; de Vivo et al., 2017). This process functions restore the total synaptic strength to a sustainable energy level, favoring sleep-dependent memory consolidation after experience-dependent synaptic potentiation during wakefulness. Specifically, slow waves (0.5–4 Hz), which may reflect the homeostatic regulation of sleep, are closely associated with mechanisms of neural plasticity (Abel et al., 2013), which is thought to be the cellular substrate underlying the processes of memory formation and consolidation. Moreover, sleep has been found to enhance neural plasticity, and REM sleep and NREM sleep spindles may promote different forms of memory consolidation, while slow-wave activity may promote Hebbian and non-Hebbian synaptic plasticity (Tononi and Cirelli, 2006).

Brain circuits in the amygdala are regulated by GABAergic neurons and other neurotransmitters to modulate stress and anxiety responses under both normal and pathological conditions (Biggio et al., 1990, 1992; Nuss, 2015). Thus, GABAergic neurons, through their inhibitory control over the hypothalamus–pituitary–adrenal (HPA) axis activity, play an important role in stress response. GABA inputs rendered ineffective to synaptic inhibition of the HPA axis via disruption of this regulatory response thereby contributing to the abnormal effects of chronic stress exposure. Recent data support the hypothesis that stress causes major changes in the GABAergic system, affecting neuroplasticity especially in the prefrontal cortex (Gilabert-Juan et al., 2013), in turn compromising emotional processing and vulnerability to stress-related disorders (Fogaça and Duman, 2019).

#### Implications for insomnia disorder pathophysiology

Low levels of GABA or impaired GABAergic transmission are associated with the etiology and maintenance of acute and chronic stress (Jie et al., 2018) and acute and chronic insomnia (Gottesmann, 2002; Plante et al., 2012).

Current evidence for the pathophysiology of insomnia converges on the hypothesis that it is a stress-related disorder according to the diathesis-stress model, with hyperarousal as a key factor (Riemann et al., 2010, 2015). In fact, evidence for increased central nervous system (CNS) brain metabolism, sympathetic activity, high-frequency EEG activation and REM density, activation of arousal promoting neurotransmission consistent with the central and peripheral hyperactivation of stress and inflammatory systems, have been shown in insomnia (Riemann et al., 2010, 2015). Insomnia is therefore considered a stressor which impairs neuroplasticity leading to a state of allostatic overload, favoring medical and psychiatric disorders (McEwen, 2003; Meerlo et al., 2009; Lo Martire et al., 2020; Palagini et al., 2021). In this framework, the hyperactivity of the HPA axis has consistently been demonstrated to be related to a functional reduction of the cortical GABAergic system and with deficient GABAergic synapses (Biggio et al., 1981; Biggio, 1983; Corda and Biggio, 1986) associated with CNS hyperarousal in insomnia (Winkelman et al., 2008; Plante et al., 2012). Increased GABA levels in subjects suffering from insomnia have been found and may reflect a potential compensatory allostatic response to chronic hyperarousal (Morgan et al., 2012).

Maladaptive changes in neuroplasticity are hypothesized in insomnia, reflecting GABAergic alterations (Salas et al., 2014). Healthy sleep facilitates neural plasticity thought to consolidate newly acquired and initially unstable memories, whereas studies in insomnia patients have shown that these processes may be impaired. In particular, insomnia is associated with a reduction in SWS (Backhaus et al., 2006; Baglioni et al., 2016; Krone et al., 2017) and diminished sleep-related consolidation of declarative and procedural memories. As impaired neuroplasticity favors the accumulation of neurotoxic proteins, neuroinflammation, and stress system alterations, insomnia is considered to be a causal factor in neurodegenerative (Shamim et al., 2019) and neuropsychiatric disorders (Palagini et al., 2019, 2020).

### Role of GABA_*A*_ receptor agonists in sleep, neuroplasticity, and brain systems modulating stress: Implications for insomnia treatment

The GABA_*A*_ receptor, a pentameric ligand-gated ion channel composed of 5 transmembrane glycoprotein subunits (two α, two β, and one γ), each characterized by different isoforms [α1–6, β1–3, and γ1–3], binds GABA at the extracellular level. Hypnotic benzodiazepines and Z-drugs bind at the interface of the α and γ subunits of the GABA_*A*_ receptor, acting as positive allosteric modulators and allowing a conformational modification of the subunit structure. This mechanism causes enhanced affinity of the binding site for GABA, thus increasing the frequency of the chloride ion channel opening and the consequent increase in the efficacy of inhibition on the CNS and brain excitability.

Benzodiazepines, developed in the 1950s, remain one of the most widely used therapeutic agents for the treatment of insomnia and anxiety (Nemeroff, 2003; Riemann et al., 2015), together with the so called *Z*-drugs that activate GABA_*A*_ synaptic transmission hence inhibiting the arousal promoting neurotransmission (Riemann et al., 2015). Studies have revealed that while the sedative effect is mediated by α1-containing GABA_*A*_ receptors, the anxiolytic-like and anti-stress-related actions are mediated by α2- and α3-containing GABA_*A*_ receptors, and the α5 subunit is mainly involved in the amnesic effect (Soh and Lynch, 2015; Sigel and Ernst, 2018). The hypnotic effect seems to be induced by the concomitant activation of α1, α2, α3, and α5 GABA_*A*_ receptor subtypes (Rudolph et al., 1999; Crestani et al., 2001; Sanna et al., 2002; Dias et al., 2005; Engin et al., 2018; Sigel and Ernst, 2018).

Most of the hypnotic benzodiazepines show a similar high affinity for the α1, α2, α3, and α5 receptor subtypes. In contrast, *Z*-drugs have a lower affinity for these subunits (Rudolph et al., 1999; Crestani et al., 2001; Sanna et al., 2002; Dias et al., 2005; Engin et al., 2018; Sigel and Ernst, 2018). For example, zolpidem does not bind the α5 subunit and its affinity for the α1 and for the α2, α3 GABA_*A*_ receptor subtypes, respectively, is approximately 10x and 100x lower that of triazolam (Sanna et al., 2002). Thus, benzodiazepines and *Z*-drugs induce a hypnotic effect by binding, albeit with different affinity, to GABA_*A*_ receptor α subunits including those mediating the anxiolytic effects and reducing neuroendocrine response to stress effects (Carrasco and Van de Kar, 2003; Kovacic and Somanathan, 2009; Rudolph and Knoflach, 2011; Engin et al., 2018).

Some hypnotics may produce more “physiological” sleep based on polysomnography, while other compounds may alter sleep architecture and may affect homeostatic sleep processes, sleep functions, and synaptic plasticity (Seibt et al., 2008). Similarly, other studies have raised the possibility that the short-acting benzodiazepines, commonly prescribed for insomnia, may facilitate circadian rhythm adaptation and sleep wake homeostasis, via a GABAergic mechanism, while others hypnotics do not (Buxton et al., 2000). Hence, differences can be found among GABAergic hypnotics in relation to sleep pattern and sleep regulation. It is possible that some compounds may facilitate more physiological sleep and sustain sleep-related neuroplasticity by regulating circadian and homeostatic sleep processes, while others may affect those processes.

Although it has been hypothesized that sleep facilitates synaptic plasticity related to memory consolidation, it is not clear how these processes are influenced by drugs commonly prescribed for insomnia. Different classes of hypnotics may impart different effects on plastic events normally occurring during sleep. It is known that hypnotics with high affinity for the α5 subunit, that produce anterograde amnesia and impair daytime cognitive functioning, could also impair synaptic plasticity (Seibt et al., 2008). Indeed, differences have been identified at a molecular level among hypnotics. In fact, triazolam may sustain neuroplasticity during sleep while *Z*-drugs, in particular zolpidem, show a potential impairment of neuroplasticity (Seibt et al., 2008; Hall-Porter et al., 2014) and of sleep-dependent memory processing (Fitzgerald et al., 2014).

Hence, hypnotics may show different effects on neural plasticity. In this framework, evidence to date does not support a causal relationship between hypnotic benzodiazepines and the development of neurodegenerative diseases while disrupted sleep may be a causal factor (DeKosky and Williamson, 2020; Salzman, 2020; Gallet et al., 2021).

It has been hypothesized that benzodiazepine may reduce the effect of stress on the CNS and reduce the risk of vulnerability to neurodegenerative diseases (DeKosky and Williamson, 2020). Accordingly, benzodiazepines by enhancing GABAergic neurotransmission markedly reduce the extensive activation of glutamate and cortisol secretion, exerting a neuroprotective effect on brain circuits from the negative action elicited by acute and chronic stressful conditions (Ferrarese et al., 1993; Carrasco and Van de Kar, 2003; Fries et al., 2006; Engin et al., 2018). Benzodiazepines may also interact with peripheral and central immune systems by suppressing cytokine secretion and microglia activation elicited by stress (Bhat et al., 2010; Ramirez et al., 2016; Bollinger et al., 2020).

Conversely, the anxiolytic-like effects of *Z*-drugs may differ among compounds used in experimental studies (Fitzgerald et al., 2014).

#### GABA_*A*_ receptors agonists: Implications for insomnia treatment

Commonly prescribed hypnotic benzodiazepines (brotizolam, lormetazepam, temazepam, triazolam) and *Z*-drugs (zolpidem, zopiclone, eszopiclone, zaleplon) are discussed in the following sections, with a focus on their effects on sleep pattern and sleep regulation, sleep-related synaptic plasticity, and stress-related brain systems.

#### Hypnotic benzodiazepines

##### Brotizolam

Brotizolam (8-bromo-6-(o-chlorophenyl)-1-methyl-4H triazolo[3,4-c]thieno[2,3-e]-1,4-diazepine), is one of the thieno-triazolo diazepine derivatives with α1-containing GABA_*A*_ receptors involvement in mediating the effects on sleep. It binds with high affinity to benzodiazepine receptor sites, is indicated as an hypnotic in the management of insomnia, with anticonvulsant, antianxiety, and muscle relaxant properties demonstrated in animal studies (Langley and Clissold, 1988). Brotizolam has an ‘intermediate’ elimination half-life of approximately 5 h (Langley and Clissold, 1988).

In clinical trials, brotizolam 0.125–0.5 mg hastened the onset of sleep and reduced the number and duration of nocturnal awakenings in clinical trials, increasing total sleep time and reducing total wake time (reviewed in Langley and Clissold, 1988). Minimal morning drowsiness and no residual impairment of psychomotor performance, respectively, were identified for dosages < 0.5 mg taken at night and for dosages within the recommended range of 0.125–0.25 mg. Indeed, side effects may include drowsiness, headache, and dizziness. Rebound insomnia has been reported following the sudden withdrawal of brotizolam (Langley and Clissold, 1988).

###### Effect on sleep pattern and sleep regulation

Brotizolam 0.5 mg taken at night may increase NREM stage 2 sleep and beta-activity, decrease alpha-activity, and reduce REM sleep in patients with insomnia, but was not effective at 0.25 mg, and it remains to be determined if SWS is altered (Saletu et al., 1983; Langley and Clissold, 1988). In an animal model, brotizolam facilitated re-entrainment of circadian rhythms (Yokota et al., 2000).Specifically, brotizolam 0.25 and 0.5 mg significantly reduced sleep onset latency, reduced nocturnal awakenings, and improved sleep quality compared with placebo in insomnia (Saletu et al., 1983; Langley and Clissold, 1988).

NREM Stage 2 sleep was also increased, particularly in patients with insomnia. REM sleep was not significantly altered in healthy volunteers who received doses of 0.25 mg at night. No alterations in the duration and frequency of REM sleep have been reported (Suzuki et al., 2003).

The activity of brotizolam (0.1, 0.3, and 0.5 mg) was studied in healthy subjects using quantitative pharmaco-EEG, psychometric and clinical evaluation. Power spectral density analysis showed that brotizolam (0.1, 0.3, and 0.5 mg), but not placebo, increased beta-activity, decreased alpha-activity, and augmented SWS activity in healthy subjects (Saletu et al., 1983). Decreased attention, concentration, psychomotor performance, and affectivity, and increased reaction time were also identified with brotizolam 0.5 mg (Saletu et al., 1983).

Brotizolam may also facilitate the re-entrainment of circadian rhythms (Yokota et al., 2000). In particular, large phase advances in hamster rhythm were induced with brotizolam injected during mid-subjective daytime (circadian time 6 or 9), but not at circadian time 0, 3, or 15. Moreover, significantly reduced expression of *Per1* and *Per2* was identified in the SCN at 1 and 2 h after injection of brotizolam. These results suggested that brotizolam may facilitate the re-entrainment of circadian rhythms (Yokota et al., 2000).

###### Effect on sleep-related synaptic plasticity

Although no studies have specifically investigated brain plasticity, the effect of brotizolam on the CNS in relation to sleep, arousal, and memory processes has been explored.

In studies conducted in animal model, brotizolam may contribute to reducing hyperarousal. In rats, intraperitoneal administration of 0.1–10 mg/kg brotizolam significantly retarded the rate of alpha-methyl-*p*-tyrosine-induced depletion of dopamine in the olfactory tubercle and retardation in nucleus accumbens and caudate nucleus. These data suggested that brotizolam inhibited dopamine turnover in the limbic forebrain and/or neostriatal dopaminergic neurons, likely through the facilitation of GABAergic action on dopaminergic nerve terminals (Ishiko et al., 1983).

Intraperitoneal or oral administration of brotizolam in rabbits also showed that brotizolam favored SWS, with high amplitudes in the neocortex. It also inhibited arousal responses by stimulating to the midbrain reticular formation and posterior hypothalamus (Kimishima et al., 1984).

Other studies conducted in animal model showed that brotizolam may affect sleep-dependent memory processes and produced anterograde and retrograde amnesia (Anand et al., 2007).

In studies conducted on sleep-dependent memory consolidation in humans, brotizolam did not appear to affect memory storage during sleep (Silva et al., 2003). In this study conducted in a small number of subjects (eight subjects), authors compared brotizolam 0.25 mg and zopiclone 7.5 mg taken at bedtime. Brotizolam did not affected morning recall of a standard word list learned before the bedtime dose, with a similar number of words recalled the following morning as placebo-treated subjects. Digit Symbol Substitution Test revealed no residual sedation by brotizolam or zopiclone. Brotizolam did not affect the morning recall compared to placebo, but subjects remembered less words under zopiclone treatment, suggesting that this drug could affect memory storage during sleep.

In summary, brotizolam does not appear to negatively affect the homeostatic process of sleep regulation, with no evidence suggesting impairment of SWS, which is believed to reflect mechanisms of neural plasticity (Abel et al., 2013). While it is possible that brotizolam does not affect processes of sleep-dependent brain plasticity, its exact role on brain plasticity remains to be determined.

###### Effect on stress-related brain systems

Although only a few studies have evaluated the effect of brotizolam on the stress system, evidence suggests that it may reduce cortisol under conditions of fear.

Specifically, brotizolam reduced fear in calves following exposure to a novel object test (Van Reenen et al., 2009). Holstein Friesian heifer calves received an intravenous injection of either a vehicle control or one of four doses of brotizolam. They were then subjected to a ‘combined’ test involving exposure to a novel environment for 5 min followed by the sudden introduction of a novel object for a further 10 min. Compared to vehicle treatment, the highest dose of brotizolam dose-dependently and significantly increased the time spent in locomotion. Findings supported the notion that and brotizolam may reduce related cortisol concentration in calves under fear conditions (Van Reenen et al., 2009).

##### Lormetazepam

Lormetazepam (3-hydroxy-benzo-1,4 diazepine) is a hypnotic, with potent agonistic action on central GABA_*A*_ receptors. It has a terminal half-life of 8–12 h and no active metabolites (Bixler et al., 1985). Lormetazepam 0.5–2.0 mg improved sleep onset latency, total sleep duration, number of awakenings in healthy subjects. Among side effects, hangover-like symptoms have been reported the morning following nocturnal doses of 2 mg and 1.5 mg, potentially disrupting information retrieval from short-term memory. CNS side effects, such as excitement and violence, have also been described (Faccini et al., 2019). A very high dependence potential of lormetazepam with drop formulation has recently increased concerns regarding its use (Costa et al., 2021).

###### Effect on sleep pattern and regulation

Lormetazepam reduces REM sleep and increases sleep spindles and K-complexes in both healthy subjects and patients with insomnia (Kubicki et al., 1987; Jobert et al., 1992; Nicholson and Pascoe, 1992). No studies were identified that assessed the effect of lormetazepam on circadian rhythms, or SWS.

###### Effect on sleep-related synaptic plasticity and on stress-related brain systems

No studies investigating the use of lormetazepam have been conducted on these topics.

##### Temazepam

Temazepam, a 1,4-benzodiazepine, is a hydroxylated metabolite of diazepam (3-hydroxydiazepam), with GABA_*A*_ receptor involvement in mediating its effects on sleep. Its half-life of about 5–11 h is relatively short, although this is longer in some subjects and in the elderly, and it has no active metabolites of clinical importance (Heel et al., 1981; Kales, 1990; Fraschini and Stankov, 1993; Morin et al., 2003). The usual dosage range of temazepam is 7.5 to 30 mg taken at or shortly before bedtime. Residual effects on morning performance with impaired psychomotor and cognitive function in the morning have been reported for the 30 mg dosage. Moreover, rebound insomnia may occur on withdrawal of temazepam treatment. The most frequently reported adverse events include gastrointestinal complaints, headache, dreams or nightmares, and residual sedation (Heel et al., 1981; Fraschini and Stankov, 1993; Morin et al., 2003).

###### Effect on sleep pattern and regulation

Temazepam may alter sleep pattern, largely decreasing SWS and REM sleep and increasing NREM stage 2 sleep, and therefore may interfere with the homeostatic regulation of sleep, but without affecting the circadian regulation of sleep.

A linear dose-response improvement in total sleep time and sleep latency was demonstrated for temazepam compared with placebo in sleep laboratory studies. Specifically, temazepam significantly increased sleep spindle duration, amplitude and density in frontal and central-posterior regions, with suppression of NREM SWS and REM sleep (Roehrs et al., 1986; Hemmeter et al., 2000; Karlsson et al., 2000; Erman et al., 2005; Arbon et al., 2015; Plante et al., 2015). There was no evidence to suggest a phase shifting effect of temazepam (Norman et al., 2001), or that temazepam altered the rates of entrainment of physiological rhythms (Donaldson and Kennaway, 1991).

###### Effect on sleep-related synaptic plasticity

No studies have been specifically conducted on brain plasticity related to temazepam. Indeed, Plante et al. (2015) conducted a high-density electroencephalography EEG study to evaluate the effects of temazepam on slow wave activity in 18 healthy adults compared to placebo. Temazepam was associated with significant decreases in slow-wave activity and incidence in healthy adults treated with temazepam 15 mg but not with placebo. Temazepam also reduced the magnitude of high-amplitude slow waves and their slopes in the first non-REM sleep episode, which was most prominent in frontal derivations.

Since SWS may reflect the homeostatic regulation of sleep and is closely associated with memory consolidation and, in general, with mechanisms of neural plasticity (Abel et al., 2013), it is possible that temazepam may impair processes of sleep-dependent synaptic plasticity.

###### Effect on stress-related brain systems

Only a few, albeit highly heterogeneous, studies have evaluated the effect of temazepam on the stress system. Nonetheless, evidence suggests that temazepam may reduce the HPA axis activity.

In a double-blind, random assignment, placebo-controlled study of six young women in the first half of their menstrual cycle, temazepam 20 mg significantly lowered plasma cortisol 40 min after ingestion, with lowered levels persisting after 3 h, and significantly raised plasma prolactin levels at 1 h after ingestion compared with placebo (Beary et al., 1983). The impact on cortisol was significant at 40 min and persisted for 3 h after oral ingestion. Prolactin was significantly raised at 1 h after ingestion (Beary et al., 1983).

In adult male Wistar rats, temazepam increased the release of vasopressin within the hypothalamic paraventricular nucleus in a dose-dependent manner (Welt et al., 2006). Temazepam blunted the stressor exposure-induced secretion of corticotropin (ACTH) in a dose-dependent manner and enhanced the intra-PVN release of vasopressin (AVP).

The authors suggested that temazepam may reduce HPA axis activity both directly, via GABA_*A*_ receptors, and indirectly, by increasing the intrahypothalamic concentrations of vasopressin.

##### Triazolam

Triazolam is a triazolobenzodiazepine with GABA_*A*_ receptor involvement in mediating its effects on sleep. Triazolam showed high efficacy as a positive modulator of GABA-elicited chloride currents with similar affinities for α1 (1.7), α2 (1.2), α3 (1.3), and α5 (1.4) receptors (Lelas et al., 2002; Sanna et al., 2002; Maubach et al., 2004; Licata et al., 2009). Its half-life in healthy subjects is relatively short (approximately 2–3 h), with hypnotic dosages from 0.125 to 0.25 mg taken at bedtime.

Triazolam is generally well tolerated, with no impairment of psychomotor or cognitive functioning the day after ingestion (Uemura et al., 2015), although residual drowsiness (‘hangover’) is dose-dependent and may occur the next morning at dosages of 1 mg or greater with the incidence usually being no greater than with a placebo after a 0.25 mg dose (5.7% for both), slightly higher after 0.5 mg (8.1%), but about four times greater after 1 mg (Takahashi et al., 2003). Side effects, including headache, dizziness, nervousness, and dry mouth, are usually mild and are reported in less than 4% of patients receiving triazolam 0.125–0.25 mg, and these have usually been mild. Insufficient data were available for meta-analysis of adverse events associated to a greater dose (0.25 mg) of triazolam (Sateia et al., 2017).

###### Effect on sleep pattern and sleep regulation

Triazolam at the therapeutic dosage may reduce stage 1 sleep and increase stage 2 sleep, with minimal effects on SWS and REM sleep at the lower dosage. Triazolam has also been shown to depress the generation of SWS without disrupting the homeostatic and ultradian processes of sleep regulation. Triazolam may also facilitate the re-entrainment of mammalian circadian rhythms, improve parameters of sleep–wake homeostasis, and sleep architecture in addition to alleviating insomnia properties. Findings in human studies support the conclusion that triazolam has chronobiotic properties (Kanno et al., 1993; Buxton et al., 2000).

In human sleep laboratory studies in healthy volunteers, triazolam 0.125 mg caused dose-dependent effects on sleep stages in both healthy volunteers and insomnia patients, significantly decreased stage 1 sleep and increased stage 2 sleep compared with baseline, with no or only minimal effects on SWS (Kanno et al., 2000). Studies showed that Triazolam may act on sleep architecture without disrupting the homeostatic and ultradian processes of sleep regulation (Borbély and Achermann, 1991). In a recent study conducted in animals, intravenous administration of triazolam (0.1 mg/kg, i.v.), zopiclone (2 mg/kg, i.v.), and zaleplon (1 mg/kg, i.v.) showed a remarkable increase in the delta wave activity (Noguchi et al., 2002), supporting early observations.

A moderate reduction in REM sleep during the first half of the night occurred after triazolam 0.25 or 0.5 mg, however, REM sleep during the first two sleep cycles was significantly reduced with triazolam 1 mg, with a compensatory increase in REM observed in the 4–6th sleep cycles (Pegram et al., 1980; Kubicki et al., 1987). In elderly patients with insomnia (60–85 years), triazolam 0.125 mg had no effect on sleep stages; % stage 1 was 22% and % stage 3–4 was 5% on both placebo and active drug.

Evidence of tolerance or rebound insomnia on abrupt withdrawal were not identified in most sleep laboratory studies, but evidence of reduced effectiveness on repeated administration was reported in one such study (Pakes et al., 1981).

Findings in animal and human studies demonstrated that triazolam can induce major shifts in the circadian clock facilitating resynchronization. Extensive studies in rodents demonstrated that triazolam phase-shifts the circadian clock (Seidel et al., 1986; Turek and Losee-Olson, 1986) and can reduce the number of days necessary for circadian rhythms to become re-entrained following 8-h shifts in the light-dark cycle (van Reeth and Turek, 1987). This phase shifting or “chronobiotic” effect of triazolam highlight the possibility that triazolam could facilitate the re-entrainment of human 24-h rhythms in addition to alleviating insomnia properties (Buxton et al., 2000; Kanno et al., 2000). For example, appropriately timed administration of triazolam facilitated adaptation of circadian rhythmicity in a human model of jet lag (Buxton et al., 2000). In this study, 6 healthy males (24–31 years old) received triazolam or placebo in two double-blind, placebo-controlled studies of an 8-h delay shift of sleep–wake and dark-light cycles simulating westward travel. Sleep recordings and 24-h cortisol and growth hormone profiles were obtained at baseline and on the first, third, and fifth days’ post-shift. With placebo, the shift induced disturbances of sleep and hormonal secretion, and a gradual re-entrainment of circadian rhythmicity was observed. On the other hand, triazolam accelerated re-entrainment of circadian rhythms (chronobiotic effect) and normalized markers of sleep/wake homeostasis significantly facilitating adaptation compared with placebo.

In summary, triazolam at the therapeutic dosage may reduce stage 1 sleep, increasing stage 2 with having minimal effects on SWS and REM sleep. If triazolam may depress the generation of NREM SWS it may do it without disrupting the homeostatic and ultradian processes of sleep regulation (Borbély and Achermann, 1991). Several studies in animals and humans showed that triazolam may facilitate the re-entrainment of human 24-h rhythms.

###### Effect on sleep-related synaptic plasticity

Available data on brain synaptic plasticity shows that triazolam may not negatively affect brain plasticity or sleep-facilitated memory consolidation in humans, with no effect on c-Fos, c-Fos gene expression is a marker of neural activity, and only a transient decrease in brain derived neurotrophic factor (BDNF) protein levels.

Triazolam had no effect on sleep-dependent brain plasticity in a canonical animal model of *in vivo* cortical plasticity (Seibt et al., 2008). The effects of triazolam, zolpidem, and ramelteon were studied on cats in the critical period of visual development. Polysomnographic recordings were performed during the entire experiment. After a baseline sleep/wake recording cats received 6 h of monocular deprivation followed by an injection of triazolam, zolpidem, ramelteon, or vehicle. They were then allowed to sleep for 8 h, after which they were prepared for optical imaging of intrinsic cortical signals and single-unit electrophysiology. Zolpidem reduced cortical plasticity by ∼50% as assessed with optical imaging of intrinsic cortical signals, while triazolam and ramelteon showed not to reduce sleep dependent brain plasticity (Seibt et al., 2008).

Since both triazolam and zolpidem bind to the α1-subunit *in vitro* (Hadingham et al., 1993) authors expected similar effects on sleep-dependent cortical plasticity. One potential explanation offered by the authors was that only zolpidem activates the α1-GABA_*A*_-receptors enough to impair plasticity (Seibt et al., 2008). Authors discussed that BDZs, like triazolam are non-selective for any subunit composition and bind with similar affinity to different GABA_*A*_-receptors subtypes explaining most of the anxiolytic effects and EEG changes (Sanna et al., 2002; Rudolph and Knoflach, 2011). In contrast, zolpidem binds to α_1_-containing GABA_*A*_-receptor with an affinity almost 10 times higher than for α_2,3–_ containing GABA_*A*_-receptors (Sanna et al., 2002; Seibt et al., 2008). Therefore, while other potential mechanisms cannot be excluded, these data can be best explained by the effect of zolpidem on GABA_*A*_-receptor subunits (Seibt et al., 2008).

Studies conducted in both humans and animals have also shown that triazolam 0.25 mg may not interfere with sleep-related memory processes. Twenty-two healthy volunteers participated in a randomized, double-blind, crossover study. All subjects received a single oral dose of zolpidem (10 mg), triazolam (0.25 mg) or placebo at 9 PM and slept for 7.5 ± 0.2 h. The effect of sleep on memory was investigated by comparing the performance of this group of volunteers with a group of 21 subjects in wakefulness condition (Meléndez et al., 2005).

Declarative memory was evaluated by using a free-recall test of ten standard word and seven no word lists. Subjects memorized lists 1 h before dosing and they were asked to recall the memorized lists 10 h after dosing. DSST was used. Neither zolpidem nor triazolam affected the enhanced non-word recall observed after sleep. None of the hypnotics affected the improvement in the DSST performance of subjects who slept (Meléndez et al., 2005).

In a study conducted in a small sample of subjects triazolam was used at higher doses than normal such as 0.375 mg and showed to adversely affect overnight motor learning (Morgan et al., 2010). This adverse effect may be due to its direct influence on motor performance, rather than interference with sleep-dependent memory consolidation. Additionally, the small sample (*N* = 12) may have provided insufficient power to detect an effect with the dose of zolpidem employed.

Another study aimed to reveal the molecular mechanisms that may contribute to the effects of benzodiazepines in the hippocampus involved in drug-related plasticity. Previous studies have demonstrated that both BDNF and c-Fos contribute to memory- and abuse-related processes that occur within the hippocampus and their expression is altered in response to BZ exposure. In a study, mice received acute or repeated administration of diazepam, zolpidem and triazolam. After acute administration of both triazolam and zolpidem there was a decreased in BDNF protein levels within the hippocampus, without any effect on c-Fos (Licata et al., 2013).

###### Effect on stress-related brain systems

Available data on the effect of triazolam on the stress system showed that triazolam may not have a negative effect on stress-induced stimulation of the HPA axis, whereas it may favor circadian regulation of 24-h cortisol and growth hormone profiles facilitating circadian adaptations in rhythmicity and sleep–wake homeostasis. By deactivating the basal forebrain and amygdaloid complexes, which are involved in the emotional regulation of anxiety and fear, of human subjects during non-REM sleep, triazolam may exert an anxiolytic effect which may be synergic to its hypnotic effect.

Although benzodiazepines are known to affect pituitary hormone release, pretreatment with triazolam or flurazepam did not significantly affect pituitary response to mild hypoglycemic stress in young healthy volunteers, with similar peaks observed in growth hormone, prolactin, and cortisol compared with placebo (Ambrosi et al., 1986). This suggests that acute benzodiazepine administration does not affect the neuroendocrine response to mild stress (Ambrosi et al., 1986). In a separate study, triazolam contributed to the circadian regulation of 24-h cortisol and growth hormone profiles, facilitating circadian adaptations in rhythmicity and sleep-wake homeostasis (Buxton et al., 2000).

A double-blind, crossover study with oxygen-15 water ([^15^O]H_2_O) Positron emission tomography (PET) has been used to study the functional neuroanatomy of human sleep, and regional cerebral blood flow (rCBF) during human sleep after administration of triazolam or placebo has been conducted. Fifteen healthy university students were studied. Using functional neuroanatomical PET, authors found that the basal forebrain and the amygdaloid complexes of human subjects showed deactivation during non-REM sleep after triazolam treatment. This is supported by the finding that the basal forebrain is deactivated during deep non-REM sleep in normal humans, suggesting that deactivation of the basal forebrain is involved in the non-REM sleep networks. The present finding showed that triazolam deactivated the basal forebrain and the amygdaloid complexes during non-REM sleep (Kajimura et al., 2004). This suggested that inhibition of the forebrain control system for wakefulness may cause the hypno inducing effect of benzodiazepines. Since the amygdaloid complexes are involved in emotional response, including anxiety and fear, the anxiolytic effect of the benzodiazepines may also be associated with their sleep-related effect (Kajimura et al., 2004).

##### *Z*-drugs

Z-drugs (zolpidem, zaleplon, zopiclone, eszopiclone) were introduced in the 1980s for the treatment of insomnia with the aim of overcoming some of the disadvantages of benzodiazepines, such as next-day sedation and daytime sleepiness, as well as dependence and withdrawal syndrome. Although initially seen as safer alternatives to benzodiazepines, concern related to the abuse and dependency of *Z*-drugs has arisen over the past three decades (Schifano et al., 2019).

##### Zolpidem

Zolpidem is an imidazopyridine with preferential affinity for the α_1_-GABA_*A*_ receptor subtype (Biggio et al., 1989; Crestani et al., 2000). Zolpidem displayed a higher affinity (*K_*i*_* = 20 nM) at α_1_-GABA_*A*_ receptors (α_1_β_2_γ_2_, α_1_β_3_γ_2_) with much lower affinity (*K_*i*_* = 400nM) at α_2_- and α_3_-GABA_*A*_ receptors (α_2_β_1_γ_2_, α_3_β_1_γ_2_) and failed to interact with GABA_*A*_ receptors containing the α_5_ subunit (α_5_β_3_γ_2_, α_5_β_2_γ_2_: *K*_*i*_≥ 20.000 nM) (Crestani et al., 2000). *In vivo* studies showed that the sedative-hypnotic and anticonvulsant effect of zolpidem were mediated by its preferential interaction with α_1_-GABA_*A*_ receptors but not with α_2_- or α_3_-GABA_*A*_ receptors (Crestani et al., 2000; Sanna et al., 2002).

The bioavailability of zolpidem is 70% in humans, with peak plasma concentration reached within 1 h of administration and an elimination half-life of 1.5–2.5 h; active metabolites are not accumulated following its degradation (Fitzgerald et al., 2014).

Increasing concern has arisen recently since zolpidem has been associated with significant increases in parasomnias, amnesia, hallucinations, and suicidality compared with placebo (Coleman and Ota, 2004; Sun et al., 2016; Wong et al., 2017). A proposed mechanism involves a pharmacodynamic interaction between serotonin reuptake inhibition and the drug (Elko et al., 1998).

Accordingly, in January 2013, the FDA recommended lower doses of zolpidem due to the risk of impaired activities that require alertness, including driving, in the morning after ingestion in some patients. The FDA informed the manufacturers that the recommended dose of zolpidem for women and aged people should be lowered from 10 to 5 mg for immediate-release products and from 12.5 to 6.25 mg for extended-release products. The FDA also informed the manufacturers that, for men, the labeling should recommend that health care professionals consider prescribing the lower doses-5 mg for immediate-release products and 6.25 mg for extended-release products. In April 2019, the FDA added a *Boxed Warning* to the prescribing information for zolpidem highlighting the risk of rare, albeit serious, injuries (including deaths) due to adverse sleep behaviors, including sleepwalking, sleep driving, and other activities while not fully awake. Common zolpidem-induced delirium and sleep-related complex behavior include accidents, falls, overdoses as well as risks to others included assaults, vehicular accidents, various crimes, and civil actions that occurred during zolpidem-induced delirium and sleep related complex behaviors (Greenblatt and Zammit, 2012; Park et al., 2016; FDA, 2019a; Harbourt et al., 2020; Westermeyer and Carr, 2020). Recently and increased concern has been developed regarding its misuse and risk of abuse (Schifano et al., 2019).

###### Effect on sleep pattern and sleep regulation

The effects of zolpidem on sleep pattern may include an increase in NREM stage 2 sleep and spindle activity, a decrease in REM and NREM SWS, with homeostatic regulation of sleep also altered. Circadian regulation of sleep has not been explored to any great extent, and no effects have been described.

Time awake after the onset of sleep was reduced after one week and increased after 2 weeks, whereas sleep latency remained reduced. Zolpidem markedly increased the duration of Stage 2 sleep without affecting either NREM SWS or REM sleep in some early studies (Monti et al., 1996). Others studies pointed out that the effects of zolpidem on sleep pattern were in the same direction of hypnotic benzodiazepines, but of smaller magnitude. Period amplitude analysis showed that the decreased SWS activity resulted mainly from a decrease in wave amplitude. SWS suppression increased with repeated drug administration, alongside increases in sigma and beta activity, spindle activity and stage 2 sleep, and decreased and/or delayed REM sleep (Feinberg et al., 2000; Kanno et al., 2000; Mednick et al., 2013).

Studies on the effect of zolpidem on circadian rhythms are limited. In one study, bedtime administration of zolpidem was evaluated in relation to circadian rhythmicity of sleep and hormones. Zolpidem (10 mg) was given to eight healthy women (aged 21–33 years) at 10.45 PM and induced a transient moderate hyperprolactinemia, but failed to alter other sleep-related hormonal secretions or endocrine markers of circadian rhythmicity (Copinschi et al., 1995).

###### Effect on sleep-related synaptic plasticity

Zolpidem may affect brain plasticity and sleep-dependent memory consolidation during sleep.

In a canonical animal model of *in vivo* cortical plasticity the effects of three different classes, benzodiazepines, imidazopyridines and melatonin receptors agonists, of hypnotics were studied on cats (male and female) in the critical period of visual development (postnatal days 28–41). Zolpidem reduced cortical plasticity by approximately 50%, which was assessed with optical imaging of intrinsic cortical signals, ramelteon minimally impaired brain plasticity, while triazolam did not affect sleep-dependent brain plasticity. Authors concluded that the effect could be explained by the binding affinity of zolpidem to the α1 subunit, although other mechanisms cannot be excluded (Seibt et al., 2008).

In humans the effect of zolpidem on sleep-dependent memory consolidation was explored. Authors compared bedtime administration of zolpidem-ER 12.5 mg and middle-of-the-night administration of zaleplon 10 mg, and placebo to 22 healthy volunteers to examine the effect of different durations of hypnotic drug exposure on memory consolidation during sleep (Hall-Porter et al., 2014).

Bedtime dosing of zolpidem-ER reduced memory testing was conducted before and after an 8-h sleep period, using a word pair association task (WPT; declarative memory) and a finger-tapping task (FTT; procedural memory). ANOVA revealed a significant negative effect for the WPT (*p* = 0.025) and a trend for the FTT (*p* = 0.067). Improvement in memory performance followed sleep compared with placebo and zaleplon, whereas placebo and zaleplon were similar. Therefore, authors concluded that hypnotic exposure with zolpidem during most of the night might negatively affect sleep-dependent memory consolidation. Results suggested that zolpidem-ER compared to zaleplon may have the potential to reduce the degree of sleep-dependent memory consolidation (Hall-Porter et al., 2014).

###### Effect on stress-related brain systems

Studies examining the effect of zolpidem on stress system are heterogeneous and no overall conclusions can be made regarding its effect. Indeed, results suggests that zolpidem may activate the HPA, or have no effect on stress-induced anxiety-like behavior, or have limited effects on pre-sleep cortisol.

In a study basal hormonal output and induction of C Fos in the hypothalamic paraventricular nucleus were measured after administration of various benzodiazepine ligands in mice. Zolpidem produced a very strong increase in plasma adrenocorticotropic hormone and corticosterone, whereas diazepam and zopiclone induced a lower increase in circulating corticosterone. Diazepam and zopiclone had no effect on corticosterone while zolpidem also appeared to activate the HPA axis by inhibiting GABAergic tonic inhibitory control on CRH neurons (Mikkelsen et al., 2005). Therefore, Zolpidem effectively stimulates the HPA axis by disinhibiting the GABAergic input onto CRH. The authors concluded that the response of benzodiazepine receptor ligands on the HPA axis is determined through the balance between pharmacological activation of α_1_- and α_2_-containing GABA_*A*_ receptors (Mikkelsen et al., 2005).

In a study the effects of three different doses (1, 3, and 10 mg/kg) of eszopiclone and zolpidem on the states of sleep and wakefulness, levels of anxiety-like behavior, and long-term contextual memory in foot shock-induced anxious rats, the administration of zolpidem at 1, 3, or 10 mg/kg doses did not attenuate stressor-induced anxiety-like behavior (Huang et al., 2010). The administration of eszopiclone at 1 mg/kg or zolpidem at 1 and 3 mg/kg doses attenuated the stressor-induced suppression of REM sleep. However, the REM sleep attenuating effects of these drugs disappeared when they were administered at higher doses. The administration of eszopiclone at 3 and 10 mg/kg doses and zolpidem at all three doses reduced the power of theta band frequencies during wakefulness. In addition, the administration of eszopiclone at 1 and 3 mg/kg doses suppressed stressor-induced anxiety-like behavior. The results of this study suggested that eszopiclone but not zolpidem may attenuated stress induced anxiety like behavior in animals (Huang et al., 2010).

To determine whether cortisol levels, both diurnal and pre-sleep, would be affected by zolpidem or placebo a study was conducted in patients with insomnia: zolpidem was found to reduce pre-sleep salivary cortisol relative to placebo but not diurnal urinary cortisol in subjects with insomnia (Roehrs and Roth, 2019). DSM-IV-TR diagnosed subjects with insomnia (*N* = 95), aged 32–70 years, having no other sleep disorder, unstable medical or psychiatric diseases or drug dependency served were studied. In this double-blind study, participants received zolpidem 10 mg or placebo for 12 months, and cortisol levels were assayed in urine and saliva samples. Higher levels of pre-sleep salivary cortisol were identified in patients with insomnia than controls, and pre-sleep cortisol was reduced with zolpidem at month 1 and 8, relative to placebo. Diurnal (0700–1500 h) urinary cortisol was not reduced with zolpidem, and was higher overall in subjects with insomnia and stable across time.

##### Zopiclone

Zopiclone is a cyclopyrrolone derivative thought to act on the GABA_*A*_ receptor complex (Wadworth and McTavish, 1993). Although zopiclone and its enantiomer, eszopiclone, are not receptor subtype-specific, zopiclone has been shown to have high affinity binding sites in the cerebral cortex, hippocampus, and cerebellum, and greater affinity for the α1 and α2 subunits than benzodiazepines, while eszopiclone has high affinity and potency for the α2 and α3 subunits (Najib, 2006; Nutt and Stahl, 2010).

Zopiclone has an elimination half-life of 3.5–6.5 h (Hajak, 1999). Zopiclone is not approved for the treatment of insomnia in United States due to its risk of abuse, and, likewise, is not available in other countries worldwide.

###### Effect on sleep pattern and sleep regulation

Zopiclone may modify sleep patterns, although studies investigating its effects on circadian sleep regulation are few and heterogeneous in nature, limiting definitive conclusions.

One study (Holmedahl et al., 2015) reported reductions in NREM 1 sleep (range, 3–8%) and increased NREM 2 sleep (range, 2–8%) within the TST in patients treated with 5 mg zopiclone vs. placebo.

Zopiclone 7.5 mg improved sleep continuity and increased stage 4 sleep in elderly healthy volunteers relative to placebo and temazepam, suggesting the specific action of zopiclone at the GABA-A benzodiazepine receptor complex may be responsible for its superior effect on sleep architecture (Hemmeter et al., 2000). Similarly, zopiclone 7.5 mg also increased deep sleep in elderly patients with insomnia (Mouret et al., 1990).

The effects of zopiclone also differed from those of classical BZDs in two studies (Mouret et al., 1990; Hemmeter et al., 2000) which found an increase in deep sleep among users of 3.5 mg zopiclone relative to placebo (Mouret et al., 1990) or temazepam (Hemmeter et al., 2000). A separate placebo-controlled study also demonstrated a decrease SWS after 6 weeks of treatment with zopiclone 7.5 mg in adults with insomnia (Sivertsen et al., 2006).

The effect of zolpidem 7.5 mg on REM sleep was assessed in two studies, which identified a reduced percentage of time spent in REM sleep (Leufkens et al., 2014) and, compared with placebo, a significant decrease in the density of REM sleep itself (Hemmeter et al., 2000) (for an overview see Louzada et al., 2021).

One study investigated the effect of zopiclone on circadian rhythms. In this study, which compared the effect of zaleplon to triazolam and zopiclone on melatonin secretion in rabbits, zopiclone did not affect plasma melatonin levels (Noguchi et al., 2003). Zaleplon increased a dose-dependent concentration of melatonin in rabbit plasma collected at 30 min after intravenous administration at doses of 1 and 2 mg/kg. In contrast, zopiclone failed to affect the plasma melatonin level.

###### Effect on sleep-related synaptic plasticity

No studies have directly investigated the effect of zaleplon on brain plasticity. Indeed, only one small study has investigated its effect on sleep-dependent memory consolidation, which showed that zopiclone may affect memory storage during sleep (Silva et al., 2003). Authors compared the effect of a single bedtime dose of brotizolam (0.25 mg) and zopiclone (7.5 mg), on memory storage in eight healthy volunteers as previously described. Brotizolam did not affect the morning recall compared to placebo, but subjects remembered significantly fewer words under zopiclone treatment, compared with placebo, suggesting that zopiclone could affect the storage of memory during sleep. Since zopiclone may modify the homeostatic process of sleep regulation impairing SWS, which is believed to reflect mechanisms of neural plasticity (Abel et al., 2013), it could be possible that zopiclone affects processes of sleep-dependent brain plasticity.

###### Effect on stress-related brain systems

Only a few studies have evaluated zopiclone in relation to the stress system. Indeed, it is uncertain whether zopiclone has an anxiolytic effect or has no effect on HPA axis.

The most widely used behavioral tests of anxiolytic properties of drugs are the conflict models. In order to test the generality of the effects observed on the plus-maze, authors compared the effects of zopiclone to those of diazepam and alprazolam on the Vogel water-lick suppression test. In the present study, it was found that zopiclone exerted an anxiolytic effect at all doses tested, qualitatively similar to those of the benzodiazepines diazepam and alprazolam (Carlson et al., 2001).

As mentioned previously, a study, which assessed the basal hormonal output measured after the administration of various benzodiazepine ligands in mice, showed that zolpidem strongly activates the HPA axis whereas diazepam and zopiclone had no effect on corticosterone (Mikkelsen et al., 2005).

##### Eszopiclone

Eszopiclone is the S-isomer of racemic zopiclone. Although eszopiclone interacts with the GABA_*A*_ receptor complex, it has a different binding profile and modulates the receptor complex in a unique manner having affinity for the GABA receptor α1 subunit as well as binding to the α2 and α3 subunits, which suggests the possibility that eszopiclone has both hypnotic and anxiolytic effects (Nutt and Stahl, 2010; Greenblatt and Zammit, 2012).

Eszopiclone is rapidly absorbed following oral administration, with peak serum concentrations at 1–1.3 h (Halas, 2006; Hair et al., 2008a,b; Rösner et al., 2018). The efficacy of eszopiclone has been evaluated in healthy adults, including elderly patients with insomnia. Compared to placebo, eszopiclone has been shown to considerably reduces sleep latency and improves sleep maintenance, duration, and quality (Halas, 2006; Hair et al., 2008a,b; Rösner et al., 2018). Accordingly, recommended clinical dosages are about 50% lower for eszopiclone compared to racemic zopiclone (Greenblatt and Zammit, 2012). The recommended starting dose for eszopiclone was initially 2 mg in non-elderly adults and 1 mg for elderly patients, (Hair et al., 2008a) but was lowered to 1 mg for all age and sex groups by a current FDA safety alert (FDA, 2014, 2019b) due to the risk of next-day impairments (Boyle et al., 2012) as shown in a randomized, double-blind cross-over study (Boyle et al., 2012) or sleep-related complex behaviors. The maximum recommended dose of eszopiclone is 3 mg in non-elderly and 2 mg in elderly subjects (Rösner et al., 2018).

###### Effect on sleep pattern and sleep regulation

In patients with insomnia, eszopiclone 1, 2, and 3 mg increased total sleep time and stage 2 sleep, but did not alter REM or SWS (Monti and Pandi-Perumal, 2007; Uchimura et al., 2012; Abad and Guilleminault, 2018). No studies have been conducted on its effect on the circadian regulation of sleep.

The hypnotic effects of eszopiclone have been described in experimental studies including a recent comparison with zolpidem on the power spectra of the EEG in the guinea pig (Ye and Garcia-Rill, 2009). This study showed that eszopiclone significantly increased NREM sleep, decreased the latency to NREM sleep, and increased the latency to REM sleep. Eszopiclone specifically increased delta band and decreased theta band activity, suggesting that delta sleep intensity was increased compared to zolpidem (Ye and Garcia-Rill, 2009).

###### Effect on sleep-related synaptic plasticity

Eszopiclone does not appear to affect synaptic plasticity or enhance adult hippocampal neurogenesis, although studies are limited to animal models.

The effect of eszopiclone on brain plasticity was studied *in vivo*. In a recent study the effects of trazodone, zaleplon, and eszopiclone in a canonical model of sleep-dependent, *in vivo* synaptic plasticity in the primary visual cortex were studied. Cats underwent 6 h of continuous waking combined with monocular deprivation to trigger synaptic remodeling. Cats subsequently received either vehicle, trazodone (10 mg/kg), zaleplon (10 mg/kg), or eszopiclone (1–10 mg/kg). Authors found that only trazodone significantly impaired sleep-dependent consolidation plasticity (Aton et al., 2009).

A sleep-related gene-expression study in mice assessed the expression of plasticity-related genes to assess synaptic plasticity changes during drug-induced sleep (Gerashchenko et al., 2017). This study found that changes in gene expression associated with synaptic plasticity can occur in the cortex in the presence of eszopiclone, suggesting that eszopiclone does not impair synaptic plasticity in the brain (Gerashchenko et al., 2017).

In another study was demonstrated that eszopiclone enhanced hippocampal adult neurogenesis in rats, suggesting that eszopiclone, presumably acting as a GABA receptor agonist, has pro-neurogenic effects in the dentate gyrus of the adult hippocampal (Methippara et al., 2010). Authors examined the effects of daily administration of eszopiclone compared with vehicle, on dentate gyrus cell proliferation and neurogenesis, and on sleep–wake patterns. Eszopiclone treatment for 7 days did not affect the rate of cell proliferation but twice-daily administration for 2 weeks increased survival of newborn cells by 46%. NREM sleep was increased on day 1, but not on days 7 or 14. Delta sleep was increased on days 1 and 7 of treatment, but not on day 14. The present study suggested that eszopiclone presumably acting as a GABA agonist, has pro-neurogenic effects in the adult neurogenesis (Methippara et al., 2010).

###### Effect on stress-related brain systems

Eszopiclone may induce anxiolytic action in a dose-dependent manner, however, further studies are needed to clarify this.

Animal studies have suggested an anxiolytic effect of eszopiclone. Specifically, eszopiclone 1 and 3 mg/kg suppressed stressor-induced anxiety-like behavior in foot shock-induced anxious rats, with a tendency for attenuating stressor-induced suppression of REM sleep (Huang et al., 2010) In addition, eszopiclone at 1 mg/kg had no effect on contextual memory, but contextual memory was significantly reduced with increased dosage. These results suggest that eszopiclone may attenuated stress-induced anxiety-like behavior in animals. Eszopiclone was also found to stimulate the HPA axis in rats, with dose-dependent increases in plasma levels of ACTH and corticosterone observed (Pechnick et al., 2011).

In another study the effects of three different doses (1, 3, and 10 mg/kg) of eszopiclone and zolpidem on the states of sleep and wakefulness, levels of anxiety-like behavior, and long-term contextual memory in footshock-induced anxious rats were explored. In addition, the administration of eszopiclone at 1 and 3 mg/kg doses suppressed stressor-induced anxiety-like behavior. The administration of zolpidem at 1, 3, or 10 mg/kg doses was not effective in attenuating stressor-induced anxiety-like behavior. Contextual memory after administration of eszopiclone at 1 mg/kg dose had no effects, but was reduced significantly with increased dosage. Contextual memory after administration of zolpidem, at all three doses, was severely disrupted. The results of this study suggested that eszopiclone but not zolpidem may attenuated stress induced anxiety like behavior in animals (Huang et al., 2010).

In another study the effects of eszopiclone on the HPA axis in the rat (Pechnick et al., 2011) was evaluated. Rats were injected with saline or eszopiclone. The acute administration of eszopiclone produced dose-dependent increases in plasma levels of ACTH and corticosterone, and tolerance developed to these effects after repeated drug administration. Pretreatment with eszopiclone did not affect stress-induced stimulation of the HPA axis. These results show that eszopiclone and the benzodiazepine-like drugs differentially affect the HPA axis (Pechnick et al., 2011).

##### Zaleplon

The pyrazolopyrimidine zaleplon (*N*-[3-(3-cyanopyrazolo[1,5-a]pyrimidin-7-yl)phenyl]-*N*-ethylacetamide) exhibits a pharmacological profile that is similar overall to that of zolpidem and acts preferentially at GABA_*A*_ receptors containing the α1 subunit (Concas et al., 1994; Sanna et al., 2002). However, the potency of zaleplon to modulate the function of GABA_*A*_ receptors containing α1, α2, or α3 subunits is lower than zolpidem (Sanna et al., 2002). Zaleplon binds to α1-containing receptors with an affinity that is 12, 10, or 27 times that apparent for its interaction with the corresponding receptors containing the α2, α3, or α5 subunits, respectively (Dämgen and Lüddens, 1999). However, the potency of zaleplon at these various receptors was one-third to one-half that of zolpidem (Sanna et al., 2002). The intrinsic activity and potency of zaleplon as an allosteric modulator of GABA_*A*_ receptor subtypes suggested that zaleplon is similar to that of the hypnotic benzodiazepines flurazepam, triazolam, and diazepam (Sanna et al., 2002). Its half-life is ultra-short, around 1 h (Terzano et al., 2003). In non-elderly and elderly patients with insomnia, zaleplon, at the usual recommended dosage of 5 and 10 mg taken at bedtime, significantly reduced sleep latency compared with placebo and 5 and 10 mg at bedtime significantly reduced sleep latency compared with placebo in clinical trials in non-elderly and elderly patients with insomnia (Terzano et al., 2003). Zaleplon 10 mg has also been shown to be free of residual hypnotic or sedative effects despite nocturnal administration as little as 2 h before waking in normal subjects compared with zolpidem (Danjou et al., 1999). In April 2019, the FDA added a *Boxed Warning* for Z-drugs, including zaleplon, advising that rare but serious injuries have happened because of sleep behaviors, including sleepwalking, sleep driving, and engaging in other activities while not fully awake (Greenblatt and Zammit, 2012; Park et al., 2016; FDA, 2019a; Harbourt et al., 2020; Westermeyer and Carr, 2020).

###### Effect on sleep pattern and sleep regulation

Zaleplon may increase SWS in the early phases, as seen in polysomnographic studies in humans, and may decrease the percentage of REM sleep (Fry et al., 2000).

Zaleplon was also shown to promote melatonin secretion in rabbits in a dose-dependent manner, with the increase in zaleplon-induced plasma melatonin levels not blocked by the benzodiazepine-receptor antagonist, flumazenil (Noguchi et al., 2003). These results may suggest that zaleplon influences chronobiology.

Zaleplon increased a dose-dependent concentration of melatonin in rabbit plasma collected at 30 min after intravenous administration at doses of 1 and 2 mg/kg. In contrast, triazolam and zopiclone failed to affect the plasma melatonin level. These results of the present studies suggest that zaleplon may promote melatonin secretion and the elevation of plasma levels of melatonin may suggest an influence of zaleplon on chronobiology (Noguchi et al., 2003).

###### Effect on stress-related brain systems

No studies have been conducted on the effect of zaleplon on the stress system.

###### Effect on sleep-related synaptic plasticity

Zaleplon may not affect brain plasticity or sleep-depended memory processes, although only a few studies have been conducted to date.

As mentioned previously, the effect of zaleplon on brain synaptic plasticity was studied (Aton et al., 2009). Authors found that while zaleplon and eszopiclone had profound effects on sleeping cortical electroencephalographic activity, only trazodone, which did not alter EEG activity, significantly impaired sleep-dependent consolidation of ocular dominance plasticity (Aton et al., 2009).

In a study conducted in humans to detect the effect of hypnotics on sleep-dependent memory consolidation, authors compared bedtime administration of zolpidem-ER 12.5 mg, zaleplon 10 mg in 22 healthy volunteers with zaleplon not affecting sleep dependent plasticity (Hall-Porter et al., 2014). The authors suggested that the drug-free period of sleep early in the night may have been sufficient for sleep-dependent memory consolidation to occur, although this hypothesis remains to be confirmed.

## Discussion

Since current models of insomnia converge on the hypothesis of insomnia as a stress–related sleep disorder with hyperarousal associated to the central and peripheral activation of the stress system, it is possible that alterations in GABAergic transmission may play a key role. When insomnia is established it is considered a stressful condition, which impairs brain plasticity leading to a state of allostatic overload favoring medical and psychiatric disorders (McEwen, 2003; Meerlo et al., 2009; Lo Martire et al., 2020; Palagini et al., 2021). Consequently, compounds to treat chronic insomnia should ideally regulate arousal, homeostatic and circadian rhythm sleep processes, the stress system, and synaptic plasticity, or, at the very least, compounds should not alter neural plasticity.

In the framework of modern insomnia models, compounds which bind the GABA_*A*_ receptor and act as positive allosteric modulators, which are already widely used in the treatment of insomnia, may still represent important compounds. Indeed, some differences have emerged at the physiological level among these compounds, which may benefit the treatment of insomnia in clinical practice.

In particular, hypnotic benzodiazepines enhance inhibitory signals to cell groups regulating arousal and inhibit arousal promoting neurotransmission (Riemann et al., 2015), and, in contrast to molecules that have a greater binding affinity to the α-1 rather than the α2 or α3 GABA_*A*_ receptors, hold an hypnoinducing effect by also binding, with similar high affinity, other GABA_*A*_ receptor α subunits including those able to reduce the neuroendocrine response to stress effects (Sanna et al., 2002; Carrasco and Van de Kar, 2003; Engin et al., 2018). This action may be extremely useful to both sleep inducing/regulating effects and in regulating overload of the stress system related to insomnia.

Among short-medium acting compounds, some differences have emerged at the molecular level. For example, among the short-medium acting benzodiazepines, triazolam, the most studied compound, may exert an important role in regulating allostasis by regulating the stress system at different levels. Moreover, by deactivating the basal forebrain and amygdaloid complexes of human subjects during non-REM sleep, triazolam may also exert an important sleep-related stress system regulation effect. On the other hand, eszopiclone, thanks to its peculiar action on α2 and α3 subunits, is emerging as a favorable compound able to exert an anxiolytic effect. However, more extensive studies are needed to clarify this topic.

Since insomnia may impair synaptic neuroplasticity it should be of importance to use, in clinical practice, compounds that are able to not additional disrupt it. In this framework, some compounds may produce more “physiological” sleep based on polysomnography while other compounds may alter sleep architecture and may affect homeostatic/circadian sleep processes, sleep functions, and consequently synaptic plasticity (Seibt et al., 2008).

Among the hypnotic benzodiazepines, brotizolam and triazolam at therapeutic dosages may reduce REM, which is increased in patients with insomnia, with no effect probably on ultradian processes of sleep regulation (Borbély and Achermann, 1991). In particular, data in human and animal studies showed that triazolam may also facilitate re-entrainment of circadian rhythms hence contributing to the regulation of sleep processes. Among *Z*-drugs, eszopiclone showed a better sleep profile, however, the regulation of circadian rhythms need to be further explored.

Indeed, it is emerging that the effect on sleep pattern may be important for sleep-dependent neural plasticity; preferentially biding α1 GABA_*A*_-receptors with hypnotic-sedative effects may impair neural plasticity, while binding with equal affinity to different GABA_*A*_ receptor subtypes may not affect this property. This has been demonstrated for triazolam in *in vivo* experimental models, which failed to impair brain plasticity during sleep compared with zolpidem (Seibt et al., 2008). Hence, among the benzodiazepines, triazolam is emerging as the compound with studies showing that its use at therapeutical doses does not impair sleep-dependent brain plasticity and may indeed be contributing to regulating sleep processes. Among *Z*-drugs, eszopiclone and zaleplon have also been shown to not affect sleep-dependent synaptic plasticity when compared with trazodone, an antidepressant used in the treatment of insomnia.

On the other hand, data on sleep-regulating effects on the stress system and synaptic plasticity are inconclusive or negative regarding temazepam, lormetazepam, zolpidem, and zopiclone. In particular, no studies have been conducted for lormetazepam on either brain plasticity or on its effect on the stress system, and great concern is emerging regarding the potential of abuse of its drop formulation. Data on zolpidem shows that it may affect sleep processes, synaptic plasticity, and sleep-dependent memory consolidation, but it may not regulate the stress system

## Conclusion

Current models of insomnia may revise the way in which we use hypnotic compounds in clinical practice. Ideally, compounds should regulate arousal, sleep processes, the stress system, and, at the very least, should not impair neural plasticity during sleep. Targeting GABA_*A*_ receptors may favor arousal control, but, depending on the compound used, sleep processes, the stress system, and synaptic plasticity may be impaired. Hypnotic benzodiazepines may be important options, because instead of producing a sedative action, they hold an hypnoinducing effect by binding with similar affinity different GABA_*A*_ receptor α subunits and by reducing neuroendocrine response to stress effects. Among them, triazolam has been the most studied compound with important effects observed on sleep processes, the stress system, and neural plasticity, which may be favorable for treating insomnia in the short term (<4 weeks). Among *Z*-drugs, eszopiclone has emerged as having a preferential profile on sleep, the stress system, and neural plasticity, although more studies are needed. In this framework, at therapeutical dosages over the short-term treatment (≤4 weeks) the benzodiazepine, triazolam, and the Z-drug, eszopiclone, may be favorable choices for the “brain” when treating patients with insomnia. Since discontinuation has been a challenge in clinical practice during the last few years, available data currently provide new opportunities to favor the use of CBT-I (Riemann et al., 2017) or the use of prolonged release melatonin 2 mg to exert a protective and chronobiotic action on the brain (for an overview see Biggio et al., 2021; Palagini et al., 2021), which must be taken into account.

## Author contributions

LP wrote the manuscript. CB supervised the final version. Both authors contributed to the article and approved the submitted version.
